# Dendritic Cells and Macrophages in the Pathogenesis of Psoriasis

**DOI:** 10.3389/fimmu.2022.941071

**Published:** 2022-06-28

**Authors:** Masahiro Kamata, Yayoi Tada

**Affiliations:** Department of Dermatology, Teikyo University School of Medicine, Tokyo, Japan

**Keywords:** dendritic cell (DC), macrophage - cell, monocyte - macrophage, langerhans cell (LC), psoriasis, psoriatic arthritis (PsA)

## Abstract

Psoriasis is a chronic inflammatory skin disease characterized by scaly indurated erythema. This disease impairs patients’ quality of life enormously. Pathological findings demonstrate proliferation and abnormal differentiation of keratinocytes and massive infiltration of inflammatory immune cells. The pathogenesis of psoriasis is complicated. Among immune cells, dendritic cells play a pivotal role in the development of psoriasis in both the initiation and the maintenance phases. In addition, it has been indicated that macrophages contribute to the pathogenesis of psoriasis especially in the initiation phase, although studies on macrophages are limited. In this article, we review the roles of dendritic cells and macrophages in the pathogenesis of psoriasis.

## 1 Introduction

Psoriasis is a chronic inflammatory skin disease characterized by scaly indurated erythema. This disease impairs patients’ quality of life enormously. Pathological findings demonstrate proliferation and abnormal differentiation of keratinocytes and massive infiltration of inflammatory immune cells. The pathogenesis of psoriasis is complicated, but it has been revealed by intensive research. Among immune cells, dendritic cells (DC) play a pivotal role in the development of psoriasis in both the initiation and the maintenance phases. In addition, it has been indicated that macrophages contribute to the pathogenesis of psoriasis especially in the initiation phase, although studies on macrophages are limited. In this article, we review the roles of DC and macrophages in the pathogenesis of psoriasis. Since the contributions of DC to the pathogenesis of psoriasis have already been well-described in the previous literature (1, 2), we give a concise overview of the current understanding. Then we review findings on the involvement of macrophages in the pathogenesis of psoriasis.

## 2 Overview of the Current Understanding of the Pathogenesis of Psoriasis and the Roles of Dendritic Cells and Macrophages

**Figure 1:**
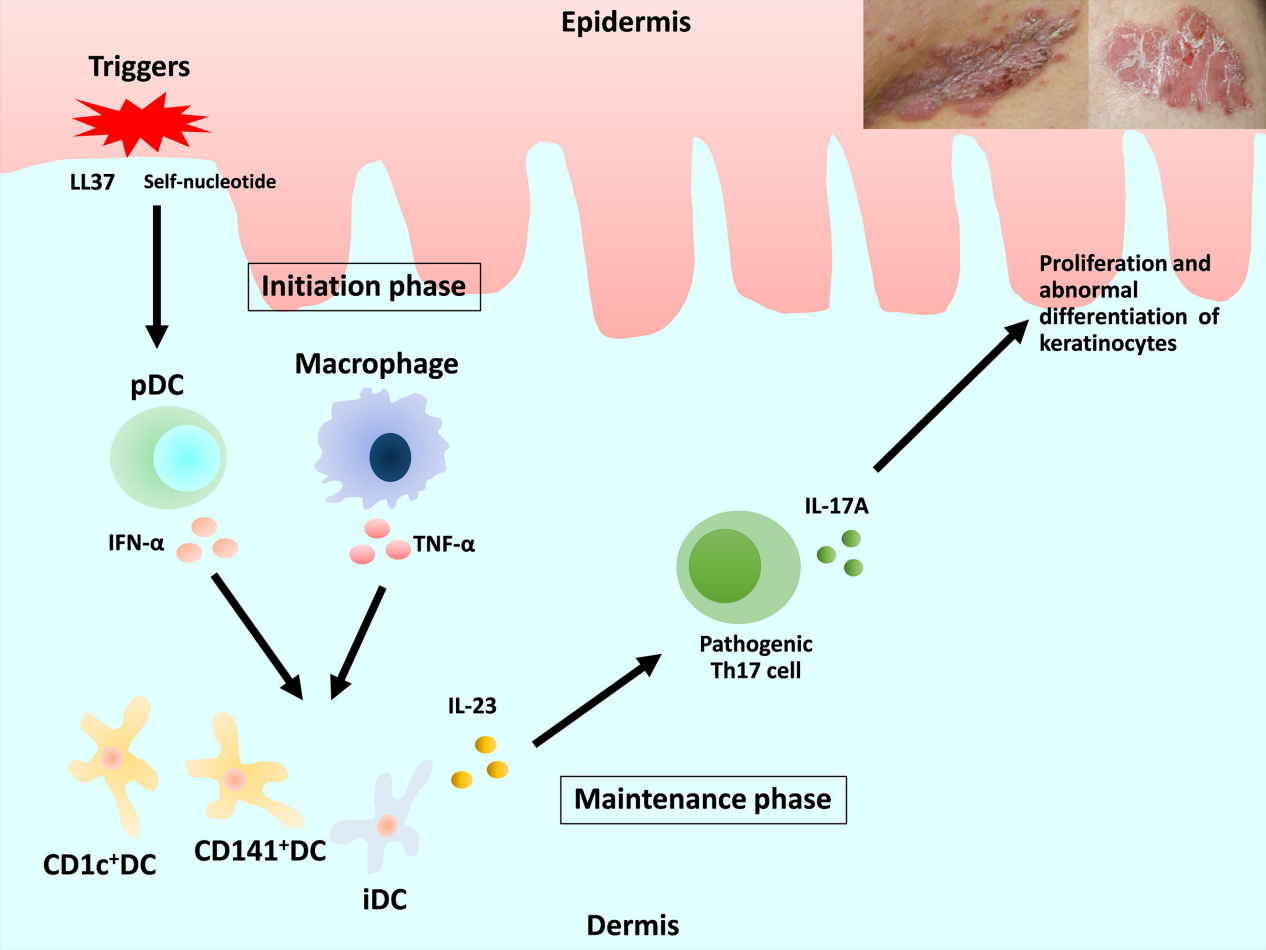
Overview of the current understanding of the pathogenesis of psoriasis and the roles of dendritic cells and macrophages. DC, dendritic cells; pDC, plasmacytoid DC; iDC, inflammatory DC.

Previous review articles have provided detailed descriptions of the pathogenesis of psoriasis (3–5). We focus on DC and macrophages (**Figure 1**). Briefly, in early-phase psoriasis, nucleic acids and a variety of antimicrobial peptides released from damaged keratinocytes activate innate immune cells, including plasmacytoid DC (pDC) and macrophages, which produce interferon (IFN)-α and tumor necrosis factor (TNF)-α. The release of IFN-α causes the maturation of resident dermal DC and the differentiation of monocytes into inflammatory DC (iDC). Mature resident DC and the rapidly increasing numbers of iDC produce interleukin (IL)-23, IL-12, TNF-α and other cytokines, which strongly activate the differentiation of naive T cells into Th1, Th17 and Th22. IL-23 maintains and promotes the proliferation of pathogenic Th17 cells. The release of IL-17 and IL-22 induces proliferation and abnormal differentiation of keratinocytes. Keratinocytes also act as immune cells by producing TNF-α, IL-8, vascular endothelial growth factor (VEGF), antimicrobial peptides, etc., some of which activate DC. This vicious inflammatory cycle causes the plaque to remain and deteriorate in the chronic phase of psoriasis (1, 2, 5, 6).

## 3 Dendritic Cells

### 3.1 Dendritic Cells Under Steady-State Conditions

DC are heterogenous and are sub-classified based on location, origin, and function. Detected subtypes of DC are different in the steady state or in the inflammatory state (6). Furthermore, there is a little difference in surface marker expressions between human DC and mouse DC (1).

In human peripheral blood, three main subsets of DC can be identified: plasmacytoid DC (pDC), and two types of conventional DC (cDC), i.e., CD1c(BDCA-1)^+^ cDC (cDC1) and CD141(BDCA-3)^+^ cDC (cDC2) (7, 8), as shown in **Table 1** (6). Hierarchical clustering of mouse lymph nodes and human blood DC subsets by genome-wide expression profiling revealed clustering of human pDC with mouse pDC, human CD1c^+^ cDC1 with mouse CD11b^+^ DC, and human CD141^+^ cDC2 with mouse CD8α^+^ DC (9).

**Table 1:** Three main subsets of dendritic cells in human peripheral blood under steady-state conditions.

Human dendritic cells	Cell surface markers on the indicated DC	Equivalent cells in mice
Plasmacytoid DC (pDC)	CD11c^+^ CD123^+^ CD303(BDCA-2)^+^ CD304(BDCA-4)^+^	Mouse pDC
Conventional DC 1 (cDC1)	CD11c^+^ CD1c(BDCA-1)^+^	Mouse CD11b^+^DC
Conventional DC 2 (cDC2)	CD11c^+^ CD141(BDCA-3)^+^	Mouse CD8α^+^DC

DC, dendritic cells.

In the skin under steady-state conditions, two dermal DC subsets identical to CD1c^+^ and CD141^+^ blood cDC have been identified (10, 11) (**Table 2**). However, pDC are absent during steady-state conditions. Human tissues also harbor migratory CD14^+^ DC, which do not have an identified murine equivalent (10, 11). Its phenotype and transcriptomic expression profiles show the characteristics of blood monocytes and tissue macrophages (10, 11), which raises the question of the origin of DC. Langerhans cells (LC) which are located in the epidermis, survey the epidermis for foreign antigens as antigen-presenting cells and activate T cells as needed (12).

**Table 2:** Human dendritic cells in the skin of normal individuals and in psoriatic skin.

Location	Human DC in the skin	Steady state	Cell surface markers in steady state*	Psoriatic skin	Cell surface markers in lesional skin**	Function in psoriasis	Equivalent cells in mice
Epidermis	LC	Present	CD11c**^+^** CD1a**^+^** CD1c**(**BDCA-1)**^+^** CD207(Langerin)**^+^**	Present	CD1a**^+^** CD1c**(**BDCA-1)**^+^** CD11c**^+^** Langerin (CD207)**^+^**	Controversial	Mouse LC
Dermis	CD1c**^+^** DC(cDC1)	Present	CD11c^+^CD1a^+^CD1c(BDCA-1)^+^	Decreased	CD1a^+^CD1c(BDCA-1)^+^CD11c^+^	Induction and proliferation of Th1/17 cells and cytokine production	Mouse CD11b^+^DC
	CD141**^+^** DC(cCD2)	Present	CD11^low^ CD1a**^+^** CD1c**(**BDCA-1)^low/int^ CD141(BDCA-3)^+^	Increased	CD11c**^+^** CD141(BDCA-3)^+^	Induction and proliferation of Th1/17 cells and cytokine production	Mouse CD103^+^DC
	pDC	Absent		Present	CD11c^-^CD123**^+^** CD303(BDCA-2)**^+^**	Production of IFN-α and activation and maturation of dermal DC	Mouse pDC
	iDC-Tip-DC- slan DC	Absent		Present	CD11c**^+^** CD206**^+^**	Production of TNF-α, iNOS, IL-23	Mouse iDC

DC, dendritic cells; LC, Langerhans cell; pDC, plasmacytoid cell; iDC, inflammatory cell.

*Cell surface markers on the indicated DC in the steady state.

**Cell surface markers on the indicated DC in lesional skin.

### 3.2 Dendritic Cells in the Skin of Psoriasis Patients

In psoriatic lesions, pDC and myeloid DC in addition to Th1/17 CD4^+^ cells are observed in the dermis (13). Dermal DC in lesional skin can be divided into three subsets: CD1c^+^ DC, CD141^+^ DC, and CD11c^+^CD1c^−^CD141^−^ inflammatory DC (iDC), including Tip-DC and 6-sulfo LacNAc DC (slanDC), as shown in **Table 2**. In inflammatory skin conditions including psoriasis, in addition to LC, CD1c^+^ DC, and CD141^+^ DC that are already present during the steady state, pDC and iDC migrate into the skin. pDC originate in the bone marrow and migrate to the skin under pathological conditions (14). The surface expression of CCR2, a chemokine receptor expressed by monocytes and required for their migration, on iDC indicates that iDC are derived from monocytes (15–17).

#### 3.2.1 Plasmacytoid DC

Increased infiltration of pDC is observed not only in lesional skin but also in non-lesional skin of psoriasis patients, compared to normal skin from healthy controls (18–21). pDC recognize self-nucleic acids, thereby initiating inflammation of psoriasis through IFN-α production (18). Antimicrobial peptides in the epidermis of psoriasis patients, including LL-37, human β-defensin (hBD)-2, hBD-3 and lysozyme, bind self-DNA/RNA fragments released by stressed or injured keratinocytes, thereby inducing activation of pDC *via* TLR7/9 (22–27). Furthermore, DNA structures containing the neutrophil serine protease cathepsin G (CatG) and the secretory leukocyte protease inhibitor (SLPI), which are detected in lesional skin of psoriasis patients, induce the production of IFN-α in pDC. pDC play a role in early psoriasis (28).

IFN-α released by pDC activates dermal resident DC, and drives their maturation (29). Moreover, IFN-α induces rapid differentiation of human monocytes into iDC and polarizes CD4^+^ T cells into Th1 and Th17 cells (30, 31). However, an anti-IFN-α monoclonal antibody failed to ameliorate plaque psoriasis in a phase I clinical trial (32), indicating that IFN-α is not important in the maintenance phase. It rather contributes to the development of psoriasis in the early phase.

#### 3.2.2 Dermal DC

IFN-α and TNF-α released by pDC, macrophages, and other cells promote maturation and activation of myeloid DC, which play an important role in the chronic phase of psoriasis. In psoriasis patients, CD11c^+^ DC are abundant in lesional skin, while there are relatively low numbers of these cells in non-lesional skin (33). Dermal DC derived from lesional skin induce proliferation of Th1 and polarization of Th17, and they are the source of IL-23 (33–40). As stated above, dermal DC in lesional skin can be divided into three subsets: CD1c**^+^** DC, CD141**^+^** DC, and iDC (**Table 2**). The number of CD1c**^+^** DC was lower in non-lesional and lesional skin of psoriasis patients than in normal skin, whereas the number of CD141**^+^** DC was higher. Lesional skin showed a considerable increase in infiltrating iDC compared with samples obtained from healthy controls (37), which mostly account for the total increase in CD11c^+^ DC in lesional skin. Both the CD1c^+^ DC and CD1c^−^ DC populations from psoriatic skin strongly induced T-cell proliferation and production of IFN-γ and/or IL-17 to the same extent (37).

CD11c^+^CD1c^−^CD141^−^ dermal iDC, including TNF-α and inducible nitric oxide synthase (iNOS)-producing DC (Tip-DC) and slanDC, have been identified in the dermis of psoriasis patients (41–43), and they seem to play a pivotal role in the pathogenesis of psoriasis (1). These iDC in psoriasis are identified as CD11c^+^CD1c^−^ DC, distinguishing them from resident cDC, and are assumed to be derived from monocytes (11, 37, 43, 44). Tip-DC express high levels of TNF-α and iNOS. TNF-α induces keratinocytes to express ICAM-1, CXCL8, and also pro-inflammatory cytokines including IL-1β and IL-6. iNOS in inflamed tissues catalyzes the production of nitric oxide (NO), which results in vasodilation of dermal blood vessels in the lesional skin of psoriasis patients (13). In addition, Tip-DC have been shown to produce high levels of IL-23 (6, 45, 46).

Through the expression of CX3CR1 and C5aR, slanDC are recruited by the increased expression CX3CL1 and C5a in psoriatic skin (42). The complete transcriptional overlap of blood slanDC with CD16^+^ monocytes indicates that skin slanDC are derived from monocytes (11, 44). As with pDC, dermal slanDC are reactive to self-RNA-LL37 complexes (42) and induce Th1/17 cells to produce IL-17, IL-22, TNF-α and IFN-γ (42, 43). In lesional skin of psoriasis patients, dermal slanDC express abundant IL-23-p19 and TNF-α (42, 47). Autocrine TNF-α stimulation of slanDC allows for higher production levels of IL-12, IL-23, IL-1β and IL-6 (48). Treatment with infliximab and dimethyl fumarate rapidly reduced the number of slanDC (1, 49, 50). Their phenotypic signatures suggest that dermal Tip-DC and slanDC represent the same inflammatory DC population although subpopulations may exist (6).

DC3 is a newly identified subset of inflammatory CD5^−^CD163^+^CD14^+^ DC (51). Recently, single-cell analysis of human skin revealed that CD14^+^ DC3 increased in psoriasis lesional skin, and they produced IL-1β and IL-23 (52), which could contribute to the pathogenesis of psoriasis.

Accumulating dermal iDC play a key role in the progression and sustenance of psoriasis by secreting large amounts of pro-inflammatory factors including iNOS, IL-23, and TNF-α (1, 6).

#### 3.2.3 Langerhans Cells

LC are antigen-presenting cells residing in the epidermis. Once they recognize an antigen, they migrate into regional lymph nodes and present antigens. A Recent study reported identification of two steady-state (LC1 and LC2) and two activated LC subsets in the epidermis of human skin and in LC derived from CD34^+^ hemopoietic stem cells (53). LC1 are characterized as classical LCs, mainly related to innate immunity and antigen processing. LC2 are involved in immune responses and leukocyte activation. LC1 remain stable under inflammatory microenvironment, whereas LC2 are prone to being activated and demonstrated elevated expression of immuno-suppressive molecules.

In the steady state, LC are continuously replaced from a resident precursor pool (54–56). However, in the inflammatory state, LC are repopulated by blood precursors (6, 57–60).

Their role in psoriasis has not yet been elucidated. The number of LCs in lesional skin of psoriasis patients was reported to be increased (61, 62), decreased (63, 64), or the same as the number of LC in control skin samples in various articles (65, 66).

The migration of LC is impaired in psoriatic patients (67, 68). Impaired LC migration in psoriasis is due to an altered keratinocyte phenotype induced by IL-17 (69).

LC play various roles in psoriasis according to previous studies. Some articles reported that LC play an anti-inflammatory role in psoriasis (53, 62, 70). In contrast, other studies indicated that LC are involved in the development of psoriasis (66, 71–74). Several studies demonstrated that LC produced IL-23 (66, 71, 72).

The discrepant data on LC are possibly due to different LC-deficient models, methods, or other factors (1). Further investigation is necessary to clarify the roles of LC in the pathogenesis of psoriasis.

The diversity of DC populations and different functions in psoriasis may be accounted for by plasticity of DC.

## 4 Macrophages

### 4.1 Roles of Macrophages Under Steady-State Conditions and Inflammatory Conditions

Macrophages are categorized into two types: tissue-resident and infiltrating macrophages (6). Tissue-resident macrophages are long-lived non-migratory cells, and play an essential role in maintaining tissue homeostasis by clearing cell debris and promoting resolution of inflammation and wound healing (75). They are potent promoters of inflammation by producing chemokines, including CCL2, CXCL1, and macrophage inhibitory factor (MIF), and cytokines such as IL-6 and TNF-α, resulting in recruitment and activation of other immune cells (76, 77). Most tissue-resident macrophages are considered to be present from birth and are self-maintaining, independently from monocytes (78–81), except intestinal macrophages (82); however, this is still controversial.

Meanwhile, infiltrating macrophages are recruited to tissues in inflammatory conditions (6). Murine studies revealed that infiltrating macrophages originate from inflammatory monocytes. Infiltrating macrophages are divided into three populations based on function, displaying a pro-inflammatory profile (originally coined “classically activated” or “M1” macrophages), a regulatory profile, or a wound-healing profile (the latter two were originally grouped under the term “alternatively activated” or “M2” macrophages), depending on the tissue context and environmental stimuli (83, 84). According to their cell surface markers, secreted cytokines and biological functions, M2 macrophages are divided into M2a, M2b, M2c, and M2d subcategories (85). M1 polarization occurs in the presence of IFN-γ, LPS, and TNF-α. Cell surface markers of M1 macrophages are CD14^++^CD16^−^, CD40, and CD68. M1 macrophages produce IL-1β, IL-6, IL-12, IL-23, MCP-1, and TNF-α. In contrast, M2 polarization occurs in response to IL-4, IL-10, and IL-13. M2 macrophages express CD14^+^CD16^++^, CD163, and CD209 on their surface. M2 macrophages produce EGF, IL-10, PDGF, TGF-β, and VEGF (86). Among M2 macrophages, M2a macrophages, activated by IL-4 or IL-13, lead to the increased expression of IL-10, TGF-β, CCL17, CCL18, and CCL22 (85). These macrophages enhance the endocytic activity, promote cell growth and tissue repairing.

### 4.2 Roles of Macrophages in the Pathogenesis of Psoriasis

Murine studies demonstrated that depletion of macrophages improved psoriasis inflammation (87–89) and reduced the levels of Th1 cytokines, including IL-1α, IL-6, IL-23 and TNF-α to normal levels (90). These results underscore that macrophages contribute to the development and maintenance of psoriatic lesions (86).

Psoriasis patients have an increased level of circulating monocytes in peripheral blood (91, 92), and they favored the M1 phenotype (93). Furthermore, a considerable number of macrophages was observed in lesional skin (94). Immunofluorescence staining revealed that CD68^+^iNOS^+^ M1 macrophages were increased and CD68^+^CD163^+^ M2 macrophages were decreased in human psoriasis lesional skin compared with skin samples from normal individuals (95). Another study demonstrated accumulation of dermal CD68^+^ macrophages that expressed TNF-α in human psoriatic plaques (96). Other studies demonstrated that the number of CD163^+^ macrophages increased in psoriatic lesional human skin, which decreased to non-lesional skin levels after an effective treatment with TNF-α inhibitors (33, 89, 97, 98). Moreover, it was indicated that CD163^+^ macrophages produce IL-23p19 and IL-12/23p40 in addition to TNF-α and iNOS in human lesional skin (97, 99). Murine experiments revealed that in skin injected with IL-23, monocytes/macrophages characterized by the strong presence of Ly6C^hi^MHC-II^hi^ cells were the dominant immune population, particularly late in the model, and showed high expression of TNF-α but not IL-17A (100). In another murine study, when peritoneal macrophages freshly isolated from resting mice were treated with IL-23, they produced large amounts of IL-17A, IL-22 and IFN-γ, and expressed a distinctive gene expression profile compared with those of M1 and M2 macrophages (101). Under the condition of abundant IL-23 in psoriasis lesional skin, some macrophages possibly produce IL-17A, IL-22 and IFN-γ in addition to TNF-α. Since macrophage are highly plastic cells (102), the diversity of macrophage populations in psoriasis may reflect a heightened cellular plasticity.

Recently, the involvement of macrophage NLRP3 inflammasome activation in psoriasis has been reported (103–105).

### 4.3 Factors That Affect Macrophage Polarization in Psoriasis

The ratio of M1 to M2a macrophage polarization was higher in psoriatic patients comparing with that in controls (93). Treatment with TNF-α inhibitors decreased M1 phenotypes according to improvement of their clinical severity scores (88, 93).

Inappropriate and excessive activation of endosomal Toll-like receptors 7, 8, and 9 (TLRs 7-9) at the psoriasis lesion plays a pathogenic role in the onset of psoriasis. Murine experiments showed that treatment with a TLR7 agonist shifted macrophages in the psoriatic lesions to a higher M1/M2 ratio. Both exogenous and endogenous TLR7-9 ligands favored M1 macrophage polarization (106).

Blocking the signaling of 4-1BBL, a member of the TNF superfamily, reduced the expression of hallmark genes of M1 macrophages such as *Tnf*, *Nos2*, and *Il23* in imiquimod-treated mice. *In vitro* experiments revealed that deficiency of 4-1BBL resulted in reduced expression of *Tnf*, *Nos2*, *Il23*, *Il6*, and *Cxcl10* in LPS-and-IFN-γ–treated macrophages (M1), whereas the expression levels of *Il10*, *Arg1*, *Fizz1*, *Ym1*, *Egr2*, and *Mrc1* (*Cd206*) were increased in IL-4–treated 4-1BBL knock-out cells, suggesting that 4-1BBL favors the M1 polarization of macrophages (107).

Response gene to complement (RGC)-32 is important for M2 macrophage polarization and phagocytic activity, and inhibits the development of M1 macrophages (108). The level of *RGCC* (the gene encoding RGC-32) mRNA was significantly lower in lesional psoriasis than in samples from normal individuals (95). Furthermore, *Rgcc* expression was significantly reduced in the lesional skin of imiquimod-induced psoriasiform dermatitis. Considering that RGC-32 participates in M2 macrophage polarization, its reduced expression in psoriatic lesions possibly contributes to skewed macrophage polarization toward the M1 phenotype (95).

IL-35, known as an anti-inflammatory cytokine (109, 110), decreased the total number of macrophages and ratio of M1/M2 macrophages in three psoriasis models: a human keratinocyte cell line (HaCaT), a keratin 14-VEGF A-transgenic mouse model, and an imiquimod-induced psoriasis mouse model (111).

Hsa_circ_0004287, one of circular RNA (circRNA), inhibited M1 macrophage activation in an N 6-methyladenosine-dependent manner in atopic dermatitis and psoriasis (112).

Increased M1 polarization was associated with higher disease severity in psoriasis, returning to baseline after successful treatment by TNF-α inhibitors (93). TNF-α blockage inhibited M1 polarization through STAT1- and IRF-1-independent pathways.

### 4.4 Factors That Affect Macrophage Recruitment to the Skin in Psoriasis

Sphingosine-1-phosphate receptor 4 (S1PR4)-dependent CCL2 production may be involved in macrophage recruitment to the psoriatic lesion (113). In imiquimod-induced psoriasiform dermatitis, psoriasis severity was ameliorated in S1PR4-deficient mice without altered IL-17 production compared with those in psoriatic wild-type mice. Instead, deficiency of S1PR4 attenuated the production of CCL2, IL-6, and CXCL1 and subsequently reduced the number of infiltrating monocytes and granulocytes. Migration assays revealed reduced CCL2 production in murine skin and attenuation of monocyte migration under the conditions lacking S1PR4. S1PR4 signaling synergized with TLR signaling in resident macrophages to produce CCL2. They speculated that S1PR4 activation enhanced the TLR response of resident macrophages to increase CCL2 production, which attracted further macrophages.

Furthermore, the importance of the interaction between CX3CL1 and CX3CR1 has been postulated in psoriasis (114). CX3CR1, a receptor for CX3CL1, mediates migration of inflammatory cells. CX3CR1 deficiency attenuated imiquimod-induced psoriasis-like skin inflammation with decreased M1 macrophages.

### 4.5 Macrophage-Specific Soluble Factors in Psoriasis

Macrophage-specific soluble factors are involved in the pathogenesis of psoriasis. Macrophages produce monocyte chemoattractant protein-1 (MCP-1), which recruits Th1 inflammatory cells (86). MCP-1 and its receptor CCR2, expressed on dermal macrophages (115), are essential for monocyte recruitment from the circulation (116). Increased expression of MCP-1 is observed in psoriatic keratinocytes (115, 117). MCP-1 polymorphisms have been associated with an increased risk of psoriasis, and serum MCP-1 levels are higher in psoriatic patients (118) and in induced psoriatic lesions of murine models (88, 94). Local production of chemotactic MCP-1 correlated with macrophage accumulation in psoriasis, suggesting that MCP-1 dysregulation may contribute to the pathogenesis of psoriasis.

Macrophage migration inhibitory factor (MIF) is another cytokine implicated in the pathogenesis of psoriasis. MIF is produced by macrophages and recruits inflammatory cells (86). MIF polymorphisms have been associated with an increased risk of psoriasis (119–121). MIF drives murine psoriasiform dermatitis (122). Serum MIF levels were higher in psoriasis patients than in healthy controls, and the serum MIF level was positively correlated with the clinical severity score. Peripheral blood mononuclear cells from psoriatic patients spontaneously produced approximately ten-fold more MIF in *in vitro* culture, indicating an inherent overproduction of this cytokine in psoriatic patients (123). In MIF-null mice, severity of psoriasiform dermatitis was lower and macrophage recruitment was impaired (122). Thus, MIF may be involved in the recruitment of macrophages in psoriasis patients. However, in contrast to elevated serum MIF in psoriasis patients, MIF-positive cells were significantly decreased in the lesional psoriatic epidermis (124). Further studies are needed to clarify the involvement of MIF in the pathogenesis of psoriasis.

### 4.6 Involvement of Macrophages in Psoriatic Arthritis

Recently, studies on macrophages in the synovial fluid (SF) of arthritic joints in patients with psoriatic arthritis (PsA) have been reported.

PsA SF cells are dominated by monocytes/macrophages. CD14^+^CD16^-^ classical monocytes/macrophages were lower in PsA SF than in the SF of patients with rheumatoid arthritis (RA), while CD14^+^CD16^+^ intermediate monocytes/macrophages were more predominant in PsA SF compared to RA SF (125). Proteinase-activated receptor 2 (PAR2) and its activating proteinases, including tryptase-6, could be important mediators of inflammation in PsA (125).

In the synovial tissues of patients with PsA and RA, synovial tissue stromal cells and CD163^+^ macrophages are the main source of granulocyte-macrophage colony-stimulating factor (GM-CSF) (126). Synovial tissue CD163^+^ macrophages express pro-inflammatory polarization markers (activin A, TNF-α, and MMP12) and exhibit a predominantly GM-CSF-dependent pro-inflammatory polarization state.

Expression of the prolactin receptor (PRLR) is higher in synovial tissue from RA and PsA patients than in synovial tissue from osteoarthritis (OA) patients, and prolactin (PRL) cooperates with other pro-inflammatory stimuli such as CD40L and TNF to activate macrophages by increasing the expression of pro-inflammatory cytokines including IL-6, IL-8 and IL-12β (127). Although serum PRL levels were similar in female and male RA patients, PRLR expression was significantly higher in RA and PsA synovial tissue compared with OA synovial tissue. PRLR colocalized with synovial CD68^+^ macrophages and von Willebrand factor^+^ endothelial cells. An *in vitro* study showed that PRLR was prominently expressed in IFN-γ- and IL-10-polarized macrophages. The production of PRL by macrophages was increased by unknown components of RA and PsA SF (128), where PRL could contribute to disease progression.

Tie2 is a tyrosine kinase receptor essential for vascular development and blood vessel remodeling through interaction with its ligands, angiopoietin-1 (Ang-1) and Ang-2 (129). Tie2 and its ligands were expressed in RA and PsA synovial tissue at higher levels than in the synovial tissue of healthy controls and OA patients (130–132). In RA and PsA synovial tissue, Tie2 was expressed by fibroblast-like synoviocytes, endothelial cells and macrophages (131, 133). Kabala et al. revealed that Tie2 signaling enhanced TNF-dependent activation of macrophages in synovial inflammation in RA and PsA patients (134).

Some of the above-mentioned factors are not specific to PsA. The role of macrophages in arthritis might be common in many aspects between RA and PsA. Although the contribution of macrophages to the development of PsA is indicated, the data are limited at present.

## 5 Conclusion

pDC play an important role in the early phase of psoriasis by producing IFN-α, which causes the maturation of resident dermal DC and the differentiation of monocytes into iDC. Increased numbers of iDC produce key cytokines of psoriasis, including IL-23, which strongly activate the differentiation of naive T cells into Th17 and Th22. IL-23 contributes to the maintenance and proliferation of pathogenic Th17 cells. The contribution of LC to the pathogenesis of psoriasis is controversial.

M1 macrophages are considered to contribute to the development of psoriasis especially in early-phase psoriasis, by producing TNF-α. Recently, IL-23 production by CD163^+^ macrophages has been reported. Further investigation is needed to clarify the involvement of macrophages in the pathogenesis of psoriasis.

## Author Contributions

MK wrote the first draft of the manuscript. YT contributed to conception, review and editing. All authors contributed to manuscript revision, read, and approved the submitted version.

## Conflict of Interest

The authors declare that the research was conducted in the absence of any commercial or financial relationships that could be construed as a potential conflict of interest.

## Publisher’s Note

All claims expressed in this article are solely those of the authors and do not necessarily represent those of their affiliated organizations, or those of the publisher, the editors and the reviewers. Any product that may be evaluated in this article, or claim that may be made by its manufacturer, is not guaranteed or endorsed by the publisher.
